# Neuronal scaffolding protein spinophilin is integral for cocaine-induced behavioral sensitization and ERK1/2 activation

**DOI:** 10.1186/s13041-019-0434-7

**Published:** 2019-02-25

**Authors:** Lorena Bianchine Areal, Alison Hamilton, Cristina Martins-Silva, Rita Gomes Wanderley Pires, Stephen S. G. Ferguson

**Affiliations:** 1 Department of Cellular and Molecular Medicine and University of Ottawa Brain and Mind Institute, University of Ottawa, 451 Smyth Road, Ottawa, ON K1H 8M5 Canada; 2 Graduate Program in Neuroscience, Institute of Biological Sciences, Federal University of Minas Gerais, Belo Horizonte, MG 31270-901 Brazil; 3 Department of Physiological Sciences, Health Sciences Center, Federal University of Espirito, Santo, Vitoria, ES 29043-910 Brazil

**Keywords:** Drug addiction, Spinophilin, Cocaine, Behavioral sensitization

## Abstract

Spinophilin is a scaffolding protein enriched in dendritic spines with integral roles in the regulation of spine density and morphology, and the modulation of synaptic plasticity. The ability of spinophilin to alter synaptic strength appears to involve its scaffolding of key synaptic proteins, including the important structural element F-actin, AMPA/NMDA modulator protein phosphatase 1, and neuromodulatory G-protein coupled receptors, including dopamine receptor D2 and metabotropic glutamate receptor 5. Additionally, spinophilin is highly expressed in the striatum, a brain region that is fundamentally involved in reward-processing and locomotor activity which receives both glutamatergic and dopaminergic inputs. Therefore, we aimed to investigate the role of spinophilin in behavioral responses to cocaine, evaluating wild-type and spinophilin knockout mice followed by the examination of underlying molecular alterations. Although acute locomotor response was not affected, deletion of spinophilin blocked the development and expression of behavioral sensitization to cocaine while maintaining normal conditioned place preference. This behavioral alteration in spinophilin knockout mice was accompanied by attenuated c-Fos and ∆FosB expression following cocaine administration and blunted cocaine-induced phosphorylation of ERK1/2 in the striatum, with no change in other relevant signaling molecules. Therefore, we suggest spinophilin fulfills an essential role in cocaine-induced behavioral sensitization, likely via ERK1/2 phosphorylation and induction of c-Fos and ∆FosB in the striatum, a mechanism that may underlie specific processes in cocaine addiction.

## Introduction

Drug addiction represents a socioeconomic and worldwide public health problem, with cocaine addiction known as one of the most prevalent drug abuse disorders [55]. Cocaine is a psychostimulant drug that blocks the reuptake of dopamine and other monoamines which primarily activates the mesocorticolimbic dopaminergic system: a key component in the reward circuitry [44]. Dopaminergic neurons from the ventral tegmental area (VTA) project to the nucleus accumbens (NAc) and dorsal striatum, as well as prefrontal cortex (PFC), hippocampus and amygdala [36, 54]. Notably, the NAc also receives glutamatergic inputs from cortical and limbic regions, such as prefrontal cortex (PFC), amygdala and hippocampus [7], thereby establishing the NAc as a converging region for dopamine and glutamate neurotransmission. While the NAc is the main region responsible for the initial rewarding effects of cocaine, the transition from voluntary to compulsive drug-use seems to involve a progressive shift towards dorsal striatum in controlling cocaine seeking behavior [10, 57, 59]. Together, corticostriatal glutamatergic and mesencephalic-striatal dopaminergic projections provide contextual information, control impulsiveness and goal-directed behavior, and regulate motivational and emotional responses to drug stimuli [13, 40].

Our laboratory has recently described the neuronal scaffold protein spinophilin as a novel Group I mGluR-interacting protein involved in the regulation of mGluR5: a prominent G protein-coupled receptor highly expressed in limbic and cortical areas [48]. Our group demonstrated that spinophilin interacts with the C-terminal tail and second intracellular loop of mGluR5, and that this interaction appears to attenuate receptor endocytosis and subsequent intracellular mGluR5-mediated ERK1/2, Akt, and Ca^2+^ signaling in primary cortical neurons [48]. Previous studies have suggested mGluR5 to have an important role in cocaine addiction, as deletion and antagonism of this receptor have been shown to decrease cocaine self-administration, cocaine-seeking after extinction, and conditioned place preference [8, 26, 28, 30]. Spinophilin has also been shown to interact with dopamine D2 receptor (D2R) via the third intracellular loop of both short and long isoforms of this receptor [51]. This receptor has also been implicated in processes underlying cocaine addiction. Altered D2R availability have been associated with high cocaine intake and compulsive use in humans and monkeys [34, 60] and conditional knockout of D2 autoreceptors in mice enhances acquisition of cocaine taking and reactivity to drug -paired cues [20].

Spinophilin is a multifunctional scaffold protein that is enriched in the dendritic spines and was first known for its interaction with phosphatase 1 (PP1α and PP1γ) and F-actin [1, 47]. Spinophilin has a role in modulating the morphology and density of dendritic spines [9, 11]. Additionally, it regulates synaptic strength by anchoring PP1 in close proximity to ionotropic glutamate receptors AMPA and NMDA and directing its substrate specificity [11, 22, 42]. Noteworthy, D1-mediated regulation of AMPA receptor activity is deficient in striatal neurons of spinophilin-KO mice [2]. Along with a PP1 binding domain and a F-actin binding site, spinophilin contains a PDZ domain, a receptor-interacting domain and a coiled-coil region [46]. Furthermore, spinophilin exhibit phosphorylation sites for several protein kinases, such as PKA, CaMKII, Cdk5 and ERK [14, 16, 21, 46, 53]. Moreover, the loss of spinophilin expression leads to impaired dopamine- and glutamate-dependent LTD, but not LTP [2, 11, 48].

Considering spinophilin’s roles in dopaminergic and glutamatergic signaling, its involvement in neuronal plasticity, and the contribution of these systems to cocaine addiction, it is foreseeable that spinophilin could play a neuromodulatory role in the mechanisms underlying cocaine addiction. Moreover, increased spinophilin expression in the prefrontal cortex of rhesus monkeys following extended cocaine self-administration has been reported, suggesting that spinophilin may be involved in the development of cocaine addiction [31]. Thus, we sought to investigate the effects of spinophilin deletion in behavioral responses to cocaine and to identify potential molecular mechanisms that may underlie its involvement.

## Materials and methods

### Material

Western blots materials and reagents were purchased from Bio-Rad. Rabbit anti–phospho-p44/42 ERK1/2 (Thr202/Tyr204), ERK1/2, phospho-GSK3β (Ser9), NMDA receptor 2A, NMDA receptor 2B, PSD95, spinophilin, tyrosine hydroxylase, phospho-Akt, mTOR, phospho mTOR, and mouse anti-GSK3β, Akt antibodies were purchased from Cell Signaling Technology. Rabbit anti-phospho tyrosine hydroxylase Ser40 was purchased from Phosphosolutions. Rabbit anti-Dopamine receptor 1, Dopamine receptor 2, c-Fos were obtained from Abcam. Rabbit anti–GAPDH (glyceraldehyde- 3-phosphate dehydrogenase) was purchased from Santa Cruz Biotechnology and rabbit anti-mGluR5 was obtained from Milipore Sigma. Antibodies validation data are available from their respective companies. For RNA extraction and qPCR Sigma-Aldrich DNase I, TRIzol reagent from ThermoFisher Scientific, iScript™ cDNA synthesis kit from Bio-Rad and Luna Universal qPCR Master Mix from New England Biolabs were used. Vector Elite ABC HRP kit (rabbit) used in immunohistochemistry was purchased from Vector Laboratories.

### Animals and drugs

Spinophilin knockout mice were generously provided by Dr. Paul Greengard (Rockefeller University, New York) and bred to a C57BL/6 background. Details on the generation of the mutant mice can be found at Feng et al., 2006. Male spinophilin knockout and wild type littermate controls (C57BL6) ageing between 8 and 12 weeks (weighing 25–32 g) were used in this study. Animals were housed in the animal care facility at the University of Ottawa on a 12-h light/12-h dark cycle, with food and water ad libitum*,* and were housed in ventilated racks with a maximum of 5 mice per cage. Experiments were conducted between 8 AM and 6 PM at the behavioural core. The order in which groups were tested was balanced between the 3 different biological replicates. All animal experiments were performed following the Canadian Council of Animal Care guidelines and approved by the University of Ottawa animal care committee (protocol no. CMM2519). Physical and behavioral well-being of animals were monitored by the Animal Care and Veterinary Service and by the experimenter. Wild-type (WT) and spinophilin knockout (KO) mice were submitted intraperitonially to 4 different treatments: saline, cocaine (15 mg/Kg), CTEP (1.5 mg/Kg) or cocaine (15 mg/Kg) + CTEP (1.5 mg/Kg) simultaneously, providing a total of 8 experimental groups. Mice were allocated to groups by simple randomization in a Microsoft Excel file and experimenters were not blinded. Sample size was based on previous studies. The drug concentrations used in this study were based on a dose response curve for locomotor activity (data not shown) and the selected doses were over the half maximum response. Although CTEP is orally bioavailable, intraperitoneal administration was chosen in order to allow for co-treatment controlling the kinetics of the drugs and to limit the potential distress of further procedures. Cocaine hydrochloride (Toronto Research Chemicals) was dissolved in sterile saline solution (NaCl 0.9%) and CTEP (Axon Biochem) was initially dissolved in sterile DMSO then diluted in saline (final DMSO concentration was 5%).

### Behavioural sensitization

**Fig. 1:**
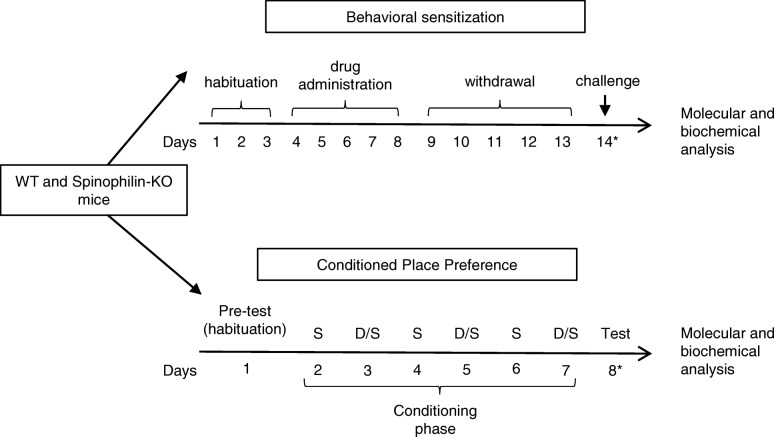
Experimental design. Wild-type and spinophilin-KO mice were submitted to either the behavioral sensitization or the condition place preference paradigm. After behavioral testing, mice had their brains dissected for biochemical assays or collected for immunostaining

Mice were tested in a square open field apparatus following a protocol described in [45]. From day 1 to 3, mice from all groups (*n* = 10–13 per group) received a saline injection and were placed in the open field arena for 30 min for habituation (Fig. 1). On days 4–8, mice from each group were injected with their respective drugs (cocaine, CTEP, cocaine+CTEP) or saline and tested in the open field for 30 min. All sessions were recorded and analyzed using the software EthoVision XT 13.0 (Noldus) for locomotor activity. After 5 days of drug administration, mice underwent a withdrawal period of 5 days followed by a challenge, where they were tested with the same drugs previously administered. Stereotypy was additionally analyzed on the challenge day. Visual analysis of stereotypic behaviors including grooming, sniffing, rearing and head bobbing was performed by a blind experimenter in 60-s observation periods every 5 min starting 10 min after injection and continuing until the end of the test as previously described [32]. In addition, stereotypic rotation behavior has been automatically analyzed through Ethovision software throughout the duration of the test (30 min).

### Conditioned place preference

CPP was assessed using a two-chamber box equipped with infrared beams (Med Associates) as previously described with modifications [38, 45]. The two compartments exhibited different patterns on the walls and floor, and were separated by a removable guillotine door. On the first day, mice were allowed to freely explore both chambers for 20 min. For the conditioning phase, mice received a saline injection and were confined to one chamber or a drug injection (cocaine, CTEP or cocaine+CTEP) and were confined to the opposite chamber for 20 min. Mice were submitted to 3 saline-paired injections and 3 drug-paired injections in alternate days, resulting in 6 days of conditioning. Conditioning was counterbalanced so that half of the mice received cocaine injections paired to side A and for the other half cocaine was paired to side B. The CPP test was performed 24 h after the last conditioning session and consisted of a drug free session where mice were allowed to freely explore both compartments for 20 min as in the habituation session. Time spent in each compartment was assessed and the data were expressed as the difference between the time spent on the drug-paired side and the saline-paired side. Ten to thirteen mice per group were tested.

### Western blotting

Mice submitted to the CPP protocol received one last injection after the test and were euthanized and dissected 10 min later for western blot analysis. This interval was chosen based on the peak of cocaine locomotor effects exhibited by these mice, potentially reflecting an optimal signalling time point. Striatum samples (from 6 to 8 animals per group) were homogenized in RIPA buffer containing protease and phosphatase inhibitors and rotated for 30 min at 4 °C. The homogenate was centrifuged at 12.000×g for 20 min and supernatant was collected for protein quantification. The lysates were diluted in SDS buffer containing 2-mercaptoethanol and 30μg of protein was separated by SDS-PAGE and transferred to a 0.45um nitrocellulose membrane. Membranes were blocked in 10% milk-TSBT solution, incubated overnight at 4 °C with primary antibodies diluted in 3% milk-TBST solution, washed, and incubated for 2 h at room temperature with secondary HRP-conjugated antibodies. For the phosphorylation assays, membranes were first blotted for phospho-protein, stripped, and then blotted for total protein. Phosphorylation levels were normalized to the total levels of the protein in interest. All antibodies used and their respective catalog numbers are listed on section 2.1 (Materials). Blots were developed with the Clarity Western ELC substrate (Bio-Rad), imaged in a Chemidoc system (Bio-Rad) and analyzed using ImageLab software (Bio-Rad).

### Gene expression analysis

qPCR was performed as previously described [3]. Striatum samples from 6 to 8 animals per group previously submitted to the CPP protocol were frozen in dry-ice immediately after dissection and stored at − 80 °C until RNA extraction. Total RNA was extracted using TRIzol Reagent (Invitrogen) as per manufacturer’s instructions. RNA samples were treated with DNase I (Sigma-Aldrich) and reverse transcribed to cDNA using iScript™ cDNA synthesis kit (Bio-rad). CFX96 Real Time PCR (Bio-rad) system and Luna® Universal qPCR Master Mix (New England BioLabs®) were used for qPCR. Relative gene mRNA expression was analyzed by 2^−ΔΔ^Ct method, using Gapdh as reference gene. All primers were validated by serial dilution and presented reaction efficiency superior than 80%. The sequences of the primers used for qPCR are presented in Table 1.

**Table 1:** Primers used for qPCR

Gene	NCBI Refseq	Sequence (5′- 3′)	Amplicon lenght (bp)
D1R	NM_010076.3	F: CCAAGAACGTGAGGGCTAAG R: TGAGGATGCGAAAGGAGAAG	120
D2R	NM_010077.2	F: CCACTCAAGGGCAACTGTACC R: TGACAGCATCTCCATTTCCAG	143
mGluR5	NM_001143834.1	F: AGTCATTTACCTAAAGCCCGG R: CTTCTCGCTGATACCCATCTG	166
NR2A	NM_008170.2	F: ATGACTATTCTCCGCCTTTCC R: AGTTTACAGCCTTCATCCCTC	220
NR2B	NM_008171.3	F: GAACGAGACTGACCCAAAGAG R: CAGAAGCTTGCTGTTCAATGG	248
DeltaFosB	NM_001347586.1	F: TGCAGCTAAGTGCAGGAACCGT R: GAGGACTTGAACTTCACTCGGCCA	224
GAPDH	NM_001289726.1	F: CCTCGTCCCGTAGACAAAATG R: TTGACTGTGCCGTTGAATTTG	194

### Immunostaining

Brain samples for immunostaining were obtained from mice submitted to the behavioral sensitization protocol that were euthanized right after the challenge test (approximately 45 min after the last drug injection). This cohort was selected for c-Fos analysis because c-Fos expression is highly detectable 30 min after stimulation in rodents [15, 65], as opposed to signalling proteins such as pERK. Briefly, 40 μm coronal sections through the striatum were performed and free-floating sections were submitted to a peroxidase-based immunostaining protocol. Sections were incubated in primary antibody for c-Fos (1:400, Abcam) overnight at 4 °C, washed, incubated in biotinylated antibody [1:400; biotinylated goat anti-rabbit IgG (Vector Elite ABC HRP kit (rabbit), Vector Laboratories], and then incubated in an avidin/biotin enzyme reagent [Vector Elite ABC HRP kit (rabbit), Vector Laboratories]. Immunostaining was visualized by reaction with a chromogen (Vector SG substrate). Sections were mounted on slides and imaged on a Zeiss LSM880 AxioObserver Z1 microscope, using representative 900 μm^2^ areas of the striatum. 3 to 5 slices from 3 brains per group were analysed.

#### Statistical analysis

Statistical comparisons were performed using GraphPad Prism 7 software. Data was analyzed by two-way ANOVA followed by Tukey *post-hoc*. For the behavioral sensitization exclusively, two-way ANOVA with repeated measures was performed. Normality was calculated by Shapiro-Wilk test to determine the appropriate use of parametric or non-parametric tests. Grubbs’ test was performed to detect outliers. Data were expressed as mean ± SEM and considered significant when *p* < 0.05.

## Results

### Spinophilin is required for cocaine-induced behavioral sensitization but not conditioned place preference

**Fig. 2:**
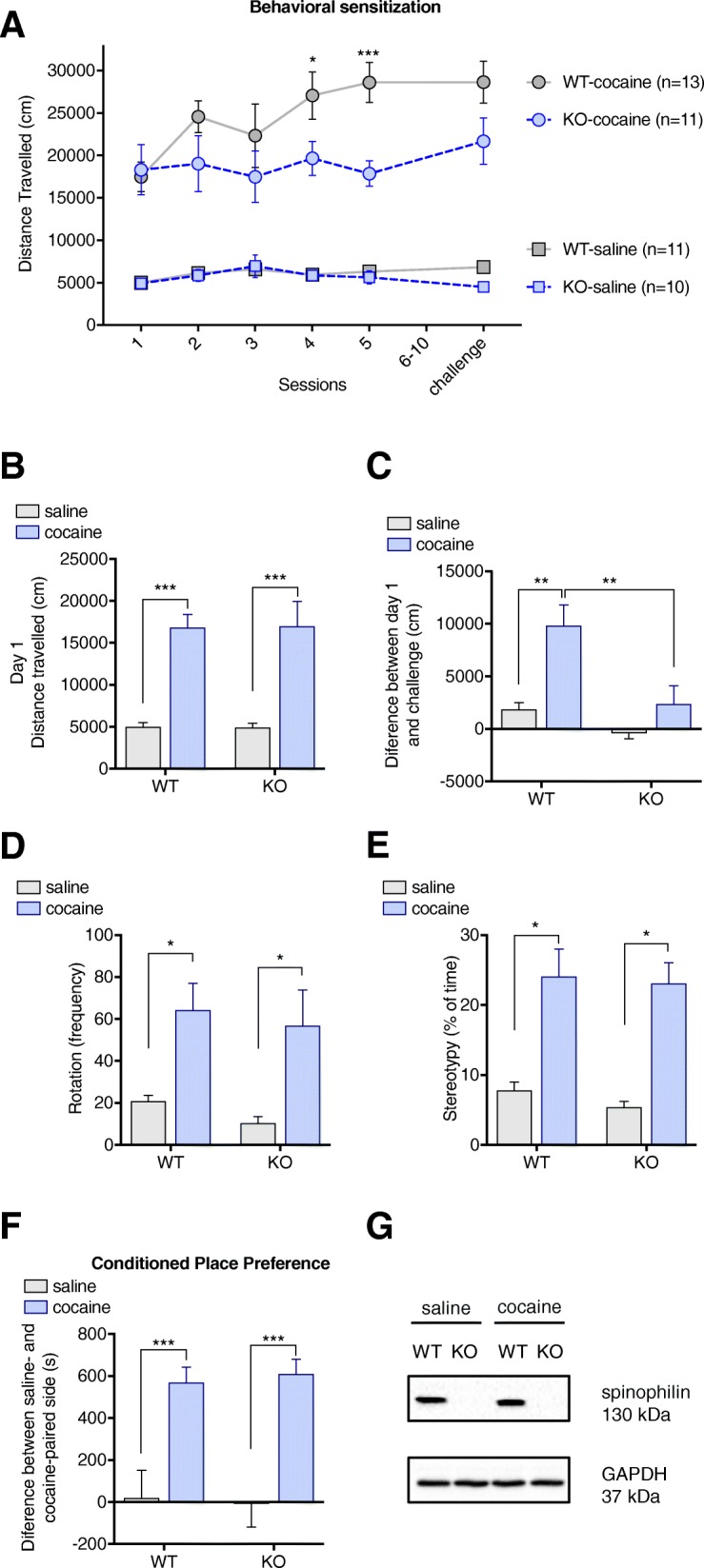
Spinophilin-KO effects on cocaine-related behaviors. **a** Locomotor responses to repeated cocaine injections in wild-type and spinophilin-KO mice. **b** Acute hyperlocomotion induced by cocaine in wild-type and spinophilin-KO mice. **c** Behavioral sensitization to cocaine treatment in wild-type and spinophilin-KO mice. **d** Stereotypic rotation behavior during the challenge test calculated by automated analysis. **e** Stereotypy (including grooming, sniffing, rearing and head bobbing) on the challenge day analyzed by a blinder experimenter. **f** Conditioned place preference to cocaine in wild-type and spinophilin-KO mice. **g** Representative western blot confirming the knockout of spinophilin. Data expressed as mean ± SEM. Two-way ANOVA with repeated measures followed by Tukey *post-hoc, n* = 10–13 per group. **p < 0.01, ***p < 0.001

A classic behavioral effect of cocaine is hyperlocomotion. Consecutive drug administrations produced different responses between WT and spinophilin-KO mice (repeated measures two-way ANOVA - interaction: F (15, 185) = 3.137 *p* = 0.0001; time effect: F (5, 185) = 4.94 *p* = 0.0003; group effect: F (3, 37) = 37.3 *p* < 0.0001) (Fig. 2a). In terms of acute locomotor responses, assessed on the first session of the behavioral sensitization protocol, there was no difference in the cocaine induced hyperlocomotion between genotypes [F (1, 40) = 44.45 *p* < 0.0001 for treatment, F (1, 40) = 0.0003448 *p* = 0.9853 for genotype, WT-cocaine vs. KO-cocaine *p* > 0.9999] (Fig. 2b). Basal locomotor activity is also not affected by deletion of spinophilin (*p* > 0.9999 for WT-saline vs. KO-saline) (Fig. 2b). While WT mice progressively increases the locomotor response to cocaine during the development phase of the behavior sensitization protocol, spinophilin-KO locomotion remained the same throughout the sessions, with significant differences between genotypes being observed on days 4 (*p* = 0.0350) and 5 (*p* = 0.0006) (Fig. 2a). In addition, Tukey’s multiple comparisons test shows a significant difference for locomotor activity between WT-cocaine and KO-cocaine on the challenge day (*p* = 0.0475). The indication of a lack of behavioral sensitization in spinophilin-KO mice is reinforced by analysis of the variation of locomotor activity on challenge day versus the first cocaine administration, with significant increase being observed only for WT mice (WT-saline vs. WT-cocaine *p* = 0.0013, KO-saline vs. KO-cocaine *p* = 0.5746, WT-cocaine vs. KO-cocaine *p* = 0.0037) (Fig. 2c). In order to verify if the lower locomotor activity observed in KO mice was possibly due to increased stereotypy induced by cocaine in this group, stereotypic behavior was analyzed on the challenge day and no difference was observed between cocaine-WT and cocaine-KO on the automated or visual analysis (WT-cocaine vs. KO-cocaine *p* = 0.9935 for stereotypies on visual analysis and *p* = 0.9621 for rotation behavior analyzed through Ethovision software). Possible differences in the rewarding effects of cocaine were also investigated, using the conditioned place preference (CPP) paradigm. However, both WT and spinophilin-KO mice developed CPP to cocaine (WT-saline vs. WT-cocaine *p* = 0.0018, KO-saline vs. KO-cocaine *p* = 0.0005) with no differences observed between genotypes (WT-cocaine vs. KO-cocaine *p* = 0.9900) (Fig. 2d).

### mGluR5 antagonism enhances locomotor activity in spinophilin-KO mice without rescuing deficits in behavioral sensitization

**Fig. 3:**
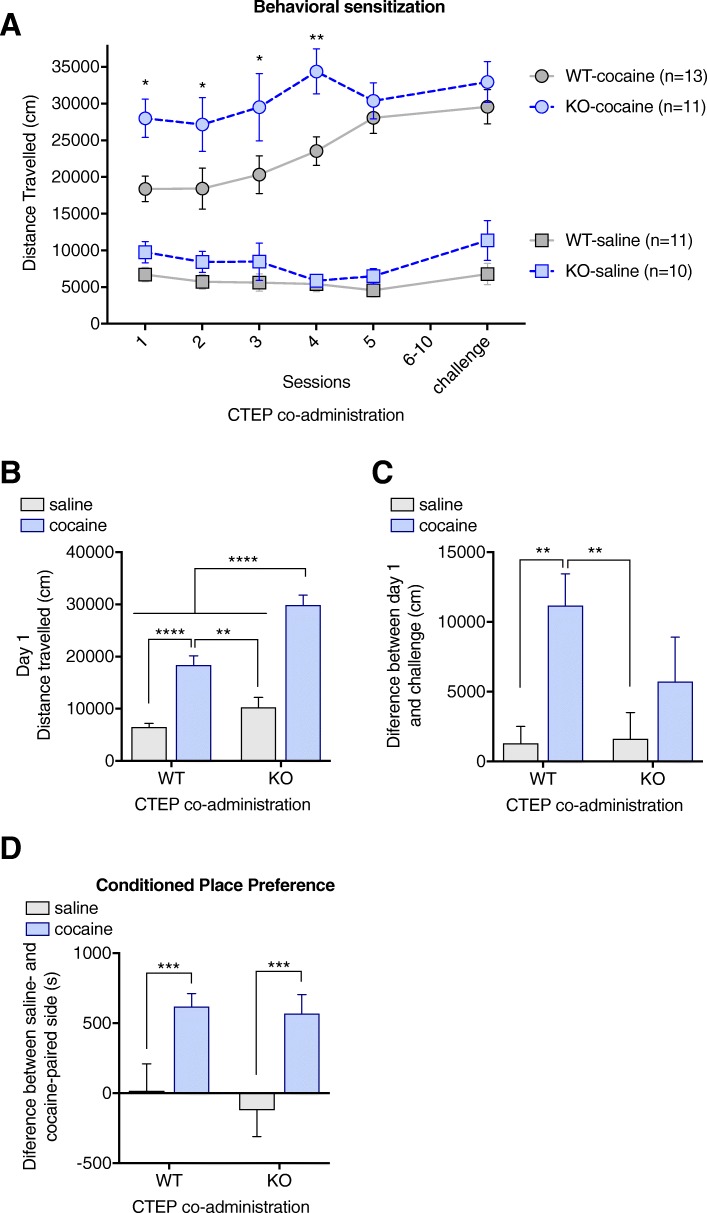
CTEP co-treatment affect acute locomotor response but not behavioral sensitization to cocaine in spinophilin-KO mice. **a** Locomotor responses to repeated co-treatment with cocaine and CTEP in wild-type and spinophilin-KO mice. **b** Acute hyperlocomotion induced by cocaine and CTEP co-treatment in wild-type and spinophilin-KO mice. **c** Behavioral sensitization to cocaine and CTEP co-treatment in wild-type and spinophilin-KO mice. **d** Conditioned place preference to cocaine and CTEP co-treatment in wild-type and spinophilin-KO mice. Data expressed as mean ± SEM. Two-way ANOVA with repeated measures followed by Tukey *post-hoc, n* = 10–13 per group. **p < 0.01, ***p < 0.001

Since spinophilin-KO mice present increased intracellular mGluR5 signalling [48] and this receptor has been implicated in the development of cocaine addiction [8, 28, 30, 66–68], we sought to investigate if a negative allosteric modulator for mGluR5 would interfere on cocaine effects in spinophilin-KO mice. Considering that there were no significant behavioral effects induced by CTEP alone (saline-WT versus CTEP-WT: *p* = 0.9988 for acute effects on first session, *p* > 0.9999 for the expression of behavioral sensitization and *p* > 0.9999 for the CPP), the data sets were separated in order to simplify results for presentation and facilitate the identification of mGluR5 involvement in the cocaine response versus spinophilin-KO effects on cocaine response. Since the first drug administration, it was observed that CTEP co-treatment potentiates cocaine-induced hyperlocomotion on spinophilin-KO mice (WT-Cocaine+CTEP vs. KO-Cocaine+CTEP *p* < 0.0001) while no difference was observed between WT and spinophilin-KO on the saline+CTEP condition (*p* = 0.4128) (Fig. 3a and b). This hyperlocomotion was sustained throughout the behavioral sensitization protocol with no further increase along the sessions (Fig. 3a). Wild-type mice that received co-administration of cocaine and CTEP exhibited normal cocaine-induced locomotor sensitization, as evidenced by an increase in the locomotor response on the challenge day versus the first administration, in comparison to CTEP control (WT-saline+CTEP vs. WT-cocaine+CTEP *p* = 0.0052) (Fig. 3c), and reached comparable locomotor activity as spinophilin-KO on day 5 (*p* = 0.8724). On the other hand, similar to previously observed, spinophilin-KO mice did not develop sensitization (KO-saline+CTEP vs. KO-cocaine+CTEP *p* = 0.6171) (Fig. 3c). However, it should be noted that the maximal locomotor response had already been achieved on the first sessions for the KO mice, which could lead to the lack of a further increase. When tested on the CPP paradigm, it was observed that mice co-administered with cocaine and CTEP developed CPP normally, independent of genotype (WT-saline+CTEP vs. WT-cocaine+CTEP *p* = 0.0465, KO-saline+CTEP vs. KO-cocaine+CTEP *p* = 0.0119, WT-cocaine+CTEP vs. KO-cocaine+CTEP *p* = 0.9945) (Fig. 3d). Co-treatment with CTEP was initially proposed to investigate if differences in cocaine effects on spinophilin-KO was mediated by mGluR5. CTEP did not affect behavioral sensitization, the striking behavioral difference promoted by deletion of spinophilin. When combined with cocaine, CTEP potentiated the hyperlocomotion, without affecting behavioral sensitization or conditioned place preference, key paradigms to study cocaine addiction in animal models. This suggests that the CTEP effect on KO mice at the dose used may be related mostly to motor aspects, in accordance with previous reports that blockage of mGluR5 produces [17, 43]. Therefore, herein we focused our molecular and biochemical studies on the cocaine effects in the presence or absence of spinophilin.

### Spinophilin deletion attenuates c-Fos and ∆FosB expression induced by cocaine

**Fig. 4:**
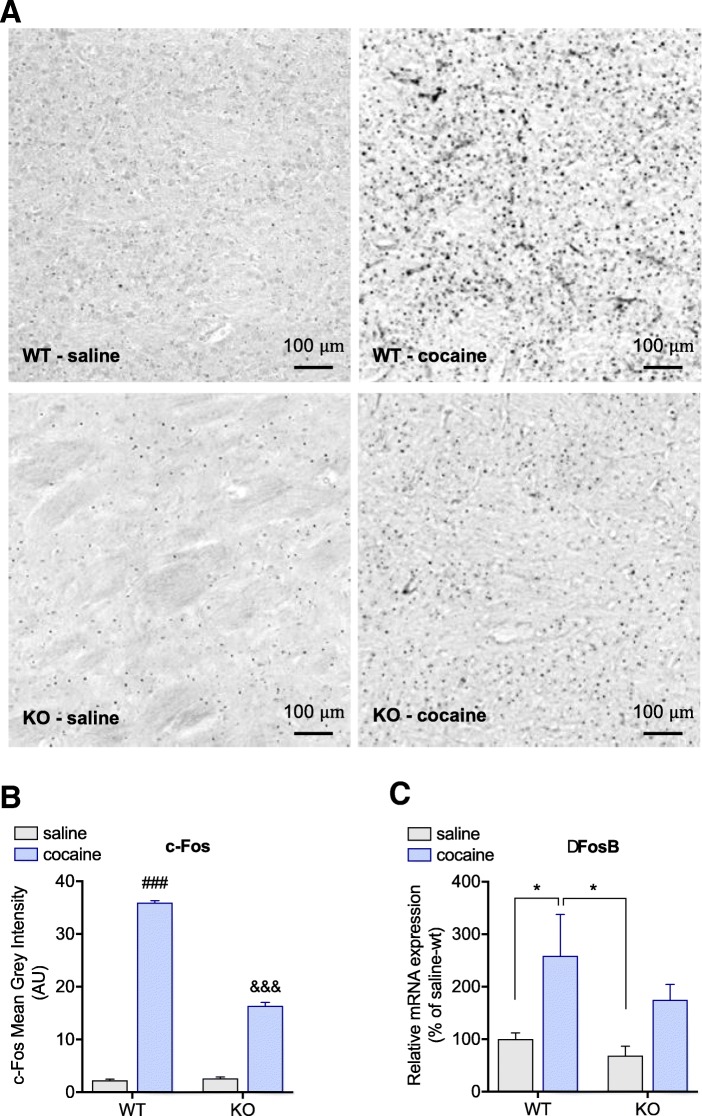
Spinophilin deletion affects cocaine induction of Fos family IEGs. **a** Representative images for immunostaining of c-Fos in the striatum of wild-type and spinophilin-KO mice. Quantification of cocaine-induced expression of **b**) c-Fos and **c**) ∆Fosb mRNA levels in wild-type and spinophilin-KO mice. Data expressed as mean ± SEM. Two-way ANOVA followed by Tukey *post-hoc*. *p < 0.05 for the indicated comparisons, ^###^*p* < 0.001 compared to all other groups and ^&&&^*p* < 0.001 compared to saline-WT and saline-KO

In order to investigate the molecular changes underlying the behavioral responses on the spinophilin-KO mice, immediate early genes from the Fos family were evaluated in the striatum of mice that underwent the behavioural sensitization protocol. Immunohistochemistry for c-Fos revealed that although both WT and KO mice administered with cocaine showed increased immunoreactivity to c-Fos (WT-saline vs. WT-cocaine *p* < 0.0001; KO-saline vs. KO-cocaine *p* < 0.0001), this effect was reduced in spinophilin-KO in comparison to WT (*p* < 0.0001) (Fig. 4a and b). Similarly, qPCR analysis of ∆Fosb showed a significant increase in mRNA levels of ∆Fosb only on WT mice (WT-saline vs. WT-cocaine *p* = 0.0479, KO-saline vs. KO-cocaine *p* = 0.2252) (Fig. 4c).

### Spinophilin-KO mice exhibit increased expression of NR2A subunit with no alterations on other main glutamate and dopamine receptors

**Fig. 5:**
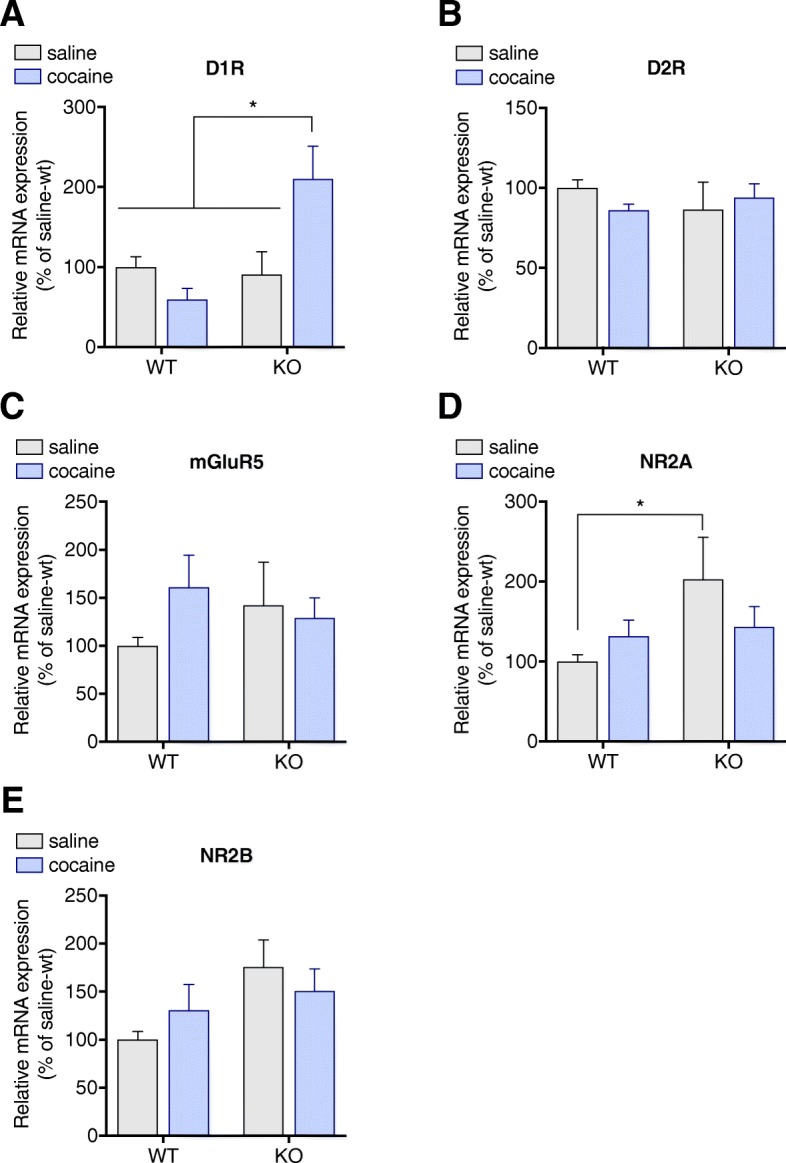
Cocaine effects on gene expression in the striatum. Cocaine-induced changes in **a**) D1R, **b**) D2R, **c**) mGluR5, **d**) NR2A and **e**) NR2B mRNA levels in wild-type and spinophilin-KO mice. Data expressed as mean ± SEM. Two-way ANOVA followed by Tukey *post-hoc*, *n* = 6–8 per group. *p < 0.05

**Fig. 6:**
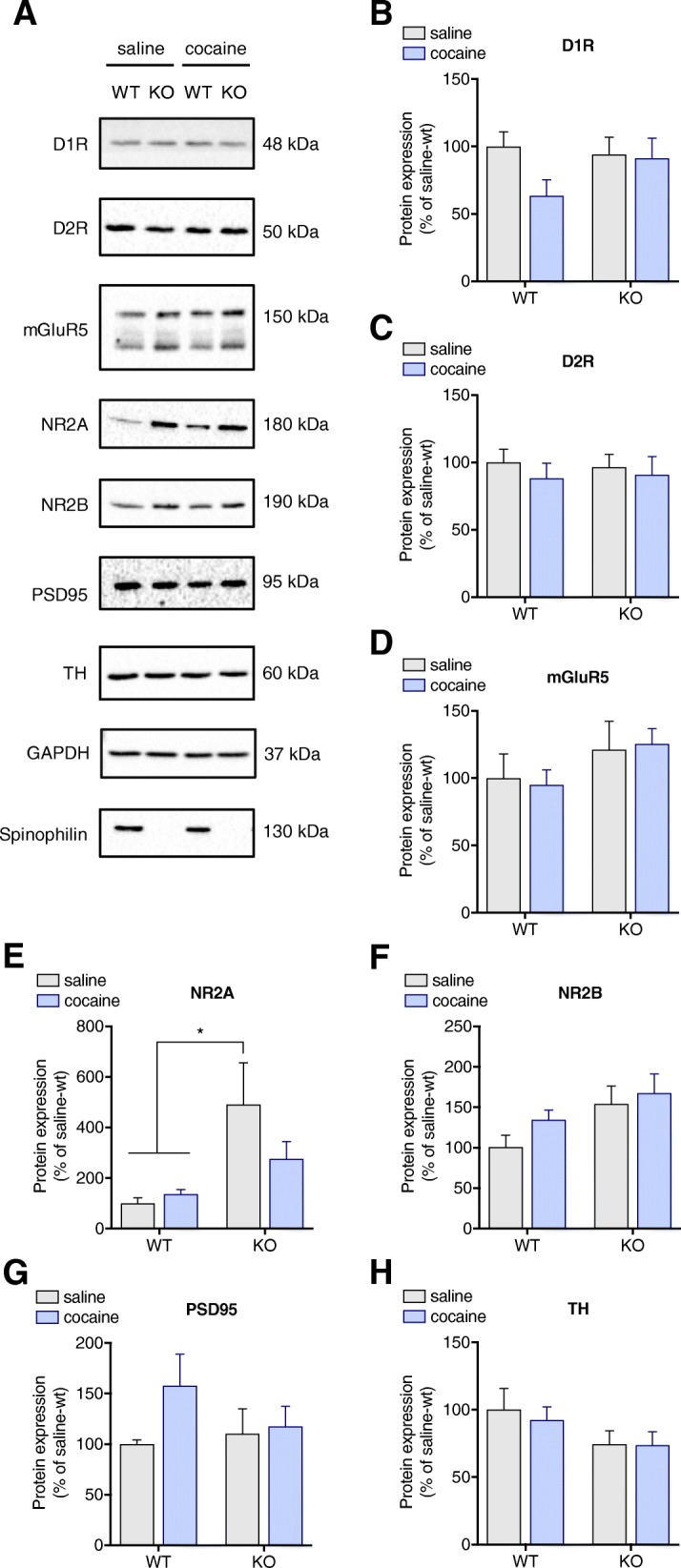
Effects of cocaine on dopamine and glutamate receptors expression in the striatum. **a** Representative images of immunoblots for D1R, D2R, mGluR5, NR2A, NR2B, PSD95, tyrosine hydroxylase (TH), GAPDH and spinophilin protein expression in the striatum of wild-type and spinophilin-KO mice. Effect of cocaine treatment on **b**) D1R, **c**) D2R, **d**) mGluR5 **e**) NR2A, **f**) NR2B, **g**)PSD95 and** h**) tyrosine hydroxylase protein expression in wild-type and spinophilin-KO mice. Data expressed as mean ± SEM. Two-way ANOVA followed by Tukey *post-hoc*, *n* = 6–8 per group. *p < 0.05

We then explored possible alterations in dopaminergic and glutamatergic signalling underlying the behavioral effects. Gene and protein expression of dopamine and glutamate receptors in the striatum were evaluated. Cocaine induced changes in D1 receptor gene expression (*p* = 0.0377) in spinophilin-KO mice only, but no changes on D2 receptor gene expression were found (Fig. 5a and b). Similarly, mGluR5 gene expression was not altered by any of the experimental treatments (Fig. 5c). Interestingly, gene expression of NMDA receptor subunit 2A was constitutively increased in spinophilin-KO mice (WT-saline vs. KO-saline *p* = 0.0289) and no significant differences were found in NR2B subunit gene expression (Fig. 5d and e). Although cocaine induced an increase in D1 receptor gene expression in the spinophilin KO mice, D1 receptor protein levels were not significantly altered (*p* = 0.9986) (Fig. 6a and b). No differences were found between treatment or genotype for either D2R protein expression (Fig. 6a and c) or mGluR5 expression (Fig. 6a and d). Similar to what was observed for gene expression of NMDA receptor subunit 2A, NMDA receptor subunit 2A protein levels in spinophilin-KO mice were also elevated in comparison to WT mice (*p* = 0.0335) (Fig. 6a and e). However, no difference between genotypes was observed after cocaine treatment (*p* > 0.05 for WT-saline vs. KO-cocaine and WT-cocaine vs. KO-cocaine). Although no significant differences on the *posthoc* were found in NR2B subunit protein levels, 2-way ANOVA showed a genotype effect (Fig. 6a and f). In order to investigate if general synaptic alterations occurred, we evaluated expression of PSD95 as a postsynaptic marker (Fig. 6g) and tyrosine hydroxylase (TH) expression as a dopaminergic presynaptic marker (Fig. 6h) and no difference was observed between groups suggesting that synapses were intact.

### Cocaine-induced activation of ERK1/2 in the striatum is absent in spinophilin-KO mice

**Fig. 7:**
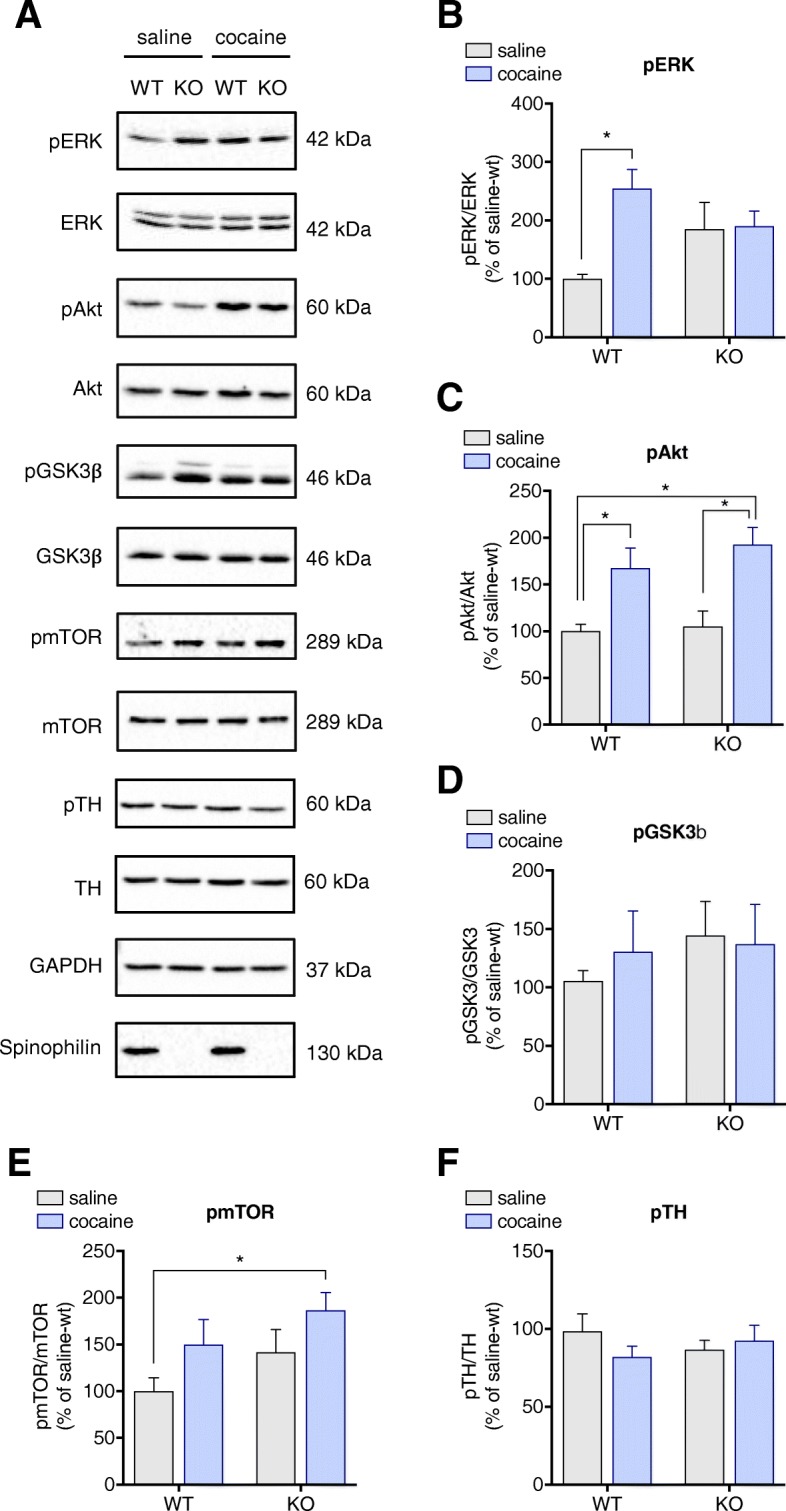
Cocaine treatment differentially affect signalling pathways in WT and spinophilin-KO mice. **a** Representative images of immunoblots for pERK, ERK, pAkt, Akt, pGSK3β, GSK3β, pmTOR, mTOR, and pTH in the striatum of wild-type and spinophilin-KO mice. Quantification of cocaine-induced changes in **b**) pERK, **c**) pAkt, **d**) pGSK3β, **e**) pmTOR, and **f**) pTH in wild-type and spinophilin-KO mice. Data expressed as mean ± SEM. Two-way ANOVA followed by Tukey *post-hoc*, *n* = 6–8 per group. *p < 0.05

To further investigate if cocaine impacts striatal signalling differently in the presence or absence of spinophilin, dopamine and glutamate signalling pathways were evaluated. Noteworthy, the increase in ERK phosphorylation induced by cocaine in the WT mice was blunted in spinophilin-KO mice (WT-saline vs. WT-cocaine *p* = 0.0077; KO-saline vs. KO-cocaine *p* = 0.9965) (Fig. 7a and b). Cocaine administration increased pAkt in both WT (*p* = 0.0385) and KO mice (*p* = 0.0049) when compared to their respective controls, with no difference found between WT-cocaine and KO-cocaine (*p* = 0.7071) (Fig. 7a and c). With respect to pGSK3β levels, no genotype or treatment effect was observed (Fig. 7a and d). Furthermore, phosphorylation of mTOR was significantly increased in spinophilin-KO mice treated with cocaine in comparison to WT-saline (*p* = 0.0429) while a trend was seen for KO-saline (Fig. 7a and e). Although not statistically significant, there was a trend for mTOR phosphorylation to be altered in the wild-type mice after cocaine treatment as well. Two-way ANOVA reveals that there is a treatment effect but not a genotype effect, in alignment with the pAkt results [pAkt/Akt: F (1, 28) = 0.8441, *p* = 0.3661 for genotype and F (1, 28) = 21.43, *p* < 0.0001 for treatment; pmTOR/mTOR: F (1, 28) = 3.218, *p* = 0.1836 for genotype and F (1, 28) = 4.688, *p* = 0.0390 for treatment]. Presynaptic signalling was also evaluated through phosphorylation levels of TH, no difference in pTH was found between the different conditions (Fig. 7a and f).

## Discussion

Spinophilin has been shown to interact with both D2R and mGluR5 which are components of dopamine and glutamate systems that are known to be involved in drug addiction. However, little is known about spinophilin’s possible role in mechanisms underlying cocaine addiction. Herein, we showed that deletion of spinophilin did not alter acute locomotor responses to cocaine, but attenuated cocaine-induced behavioral sensitization. Similar results have been reported by Morris and colleagues, where spinophilin-KO mice did not develop locomotor sensitization to amphetamine, while no genotype effect was seen on the first amphetamine administration [33]. Despite the effect of spinophilin deletion on the behavioral sensitization paradigm, this protein does not seem to be required for cocaine-induced CPP, as evidenced by the development of conditioned place preference in both wild-type and spinophilin-KO mice. A previous study suggested that spinophilin-KO mice would be more sensitive to cocaine effects based upon observations that cocaine induced CPP at a low dose (5 mg/Kg) that did not induce responses in wild-type mice. However, they observed no difference in response between genotypes at 10 mg/Kg of cocaine: a dose closer to that used in the present study (15 mg/Kg). In further comparison, spinophilin-KO mice in our study did not develop cocaine sensitization, while Allen et al. [2] report that this behavior was preserved in spinophilin-KO mice in their study. This discrepancy is likely related to important differences in the protocol used for the sensitization paradigm, since the protocol used by Allen et al. [2] consisted of 5 consecutive days where the locomotor activity was monitored for 10 min after cocaine administration (20 mg/Kg). The absence of withdrawal and challenge, shorter observational time, and higher dose in comparison to our parameters may have impacted the contrasting findings observed between the two studies.

Enhanced cocaine-induced c-Fos expression in spinophilin-KO mice has also been previously reported by Allen et al. [2]. Conversely, we observed attenuated c-Fos and ∆Fosb expression in cocaine-KO mice compared to cocaine-WT. These differences may rely on the experimental approaches, since in this study the analysis was performed after the challenge of the behavioral sensitization protocol, while in [2] it was after an acute administration, in addition, the method used for c-FOS analysis was in situ hybridization in [2], while herein immunostaining was performed. Rapid induction of Fos family proteins is a classic cellular response to cocaine and D1 agonists, especially in regions involved with the rewarding and locomotor effects such as the nucleus accumbens, dorsal striatum and prefrontal cortex [15, 29, 35]. While c-Fos returns to basal levels within hours after drug administration, ∆fosb, a very stable truncated form, accumulates with chronic exposure and can persist in the neurons for weeks after cessation of drug administration and has been shown to increase sensitivity and drug-seeking behavior to cocaine [25, 37]. Fos gene deletion in D1R-expressing neurons not only disrupts the cocaine-induction of c-Fos and ∆Fosb expression but also attenuates behavioral sensitization in the mutant mice [63]. On the other hand, normal CPP and increased extinction time were exhibited by the Fos-KO mice [63]. In accordance with that, spinophilin-KO mice, in the present study, did not develop behavioral sensitization and presented attenuated cocaine-induction of c-Fos and ∆Fosb, while CPP was maintained.

c-Fos and ∆Fosb expression can be induced by transcription factors that are activated by pERK, such as CREB and Elk-1 [49, 58, 64]. In the present study, ERK phosphorylation was not induced by cocaine treatment in the spinophilin-KO mice. Several studies indicate that ERK activity has a role in the development of cocaine-induced locomotor sensitization. It has been shown that systemic injections of MEK inhibitors during the development phase prevents the expression of cocaine-induced locomotor sensitization after withdrawal [12, 41, 56]. On the other hand, MEK inhibitors show no significant effect on either spontaneous locomotor activity or acute cocaine-induced locomotion in the cited studies. Herein, while the behavioral sensitization was absent in spinophilin-KO mice they still developed a place preference for cocaine. Although it has been shown that ERK activation is linked to both sensitization and conditioned place preference, there are several signalling pathways involved in both of these processes. For example, Akt/GSK3/mTOR signalling pathway in the nucleus accumbens, hippocampus, and/or prefrontal cortex is critically involved in the expression and reconsolidation of cocaine conditioned place preference [4, 50]. In the present study, Akt and mTOR phosphorylation in response to cocaine remained intact. Therefore, it is possible that other signaling pathways involved the development of conditioned place preference may have compensated the reduced ERK activation and been sufficient for the expression of this behavior, but not for the behavioral sensitization.

The mechanisms underlying cocaine-induced behavioral sensitization are not fully elucidated. However, the literature indicates that this phenomenon is associated with an increase in dopaminergic neurotransmission, with increases in dopamine release [24, 39] and D1R sensitivity [18, 24], as well as decreased autorreceptor activity [19, 52] and LTP induction [5] being reported. In addition, studies show that a reduction in D2R activity in the prefrontal cortex occurs in association with the development of cocaine sensitization and that repeated administrations of a D2R antagonist promotes sensitization [6, 52]. Moreover, an increase in the excitability of VTA neurons during the development of sensitization to stimulants has been associated with a decrease in the autoreceptor function of D2R [19, 52]. It is known that spinophilin binds to the third intracellular loop of D2R. However, the consequences of this interaction have not yet been elucidated as opposed to other receptors (i.e. α2AR, mu-opioid and mGluR5). Considering the structural similarity in the third intracellular loop of D2R and α2AR, and that both receptors are coupled to Gi, it could be hypothesized that spinophilin may regulate D2R in a similar manner and antagonize beta-arrestin 2- dependent MAPK signalling [62]. If that was the case, D2R signalling would be exacerbated in spinophilin-KO mice, which could be a possible mechanism underlying the prevention of behavioral sensitization observed in these mice. However, specific studies on spinophilin’s role on D2R signalling are needed in order to confirm that hypothesis.

Interestingly, we observed that spinophilin-KO mice have constitutively higher expression of NR2A. It is known that stimulation of NR2A activates ERK pathway, an important component of NMDAR signal transduction involved in synaptic plasticity [27, 61]. Notably, despite increased NR2A, ERK activation following cocaine administration was not observed in spinophilin KO mice, suggesting that spinophilin is required for the regulation of this signalling pathway. Noteworthy, [69] demonstrated that injections of D-serine, an endogenous co-agonist of NMDARs, in the NAc, blocked the behavioral sensitization to cocaine and suggested that the inhibition of ERK-CREB-Fos pathway was involved in this process [69]. The increased expression of NR2A in spinophilin-KO mice observed in this study also raised the possibility that the increased basal levels of intracellular Ca^2+^, pAkt and pERK observed in neuronal cultures from spinophilin-KO mice by [48] may be also mediated by an NR2A overexpression. However, it should be noted that results reported on [48] were obtained from cortical neurons while herein we analyzed striatum samples.

Our laboratory has previously described spinophilin’s involvement in the regulation of mGluR5, a metabotropic glutamate receptor highly expressed in limbic and cortical areas [48]. Previous studies have suggested a role for mGluR5 in cocaine addiction, as deletion and antagonism of this receptor have been shown to decrease cocaine self-administration, cocaine-seeking after extinction, and conditioned place preference [8, 26, 28]. McGueehan and Olive (2003) tested the effect of MPEP, an mGluR5 antagonist, on the rewarding properties of different drugs of abuse. The authors report that only CPP to cocaine was affected, while amphetamine, morphine, nicotine or ethanol CPP remained unaltered [30]. On the other hand, Fowler et al. (2011) showed that mGluR5 knockout mice developed normal CPP to cocaine and cocaine-induced hyperlocomotion on the same level as wild-types at moderate doses of cocaine (10 and 20 mg/Kg) [70]. In addition, a study using the mGluR5 antagonist MTEP observed no effect on cocaine CPP in three different doses tested [59]. Therefore, the literature on the potential therapeutic effect of mGluR5 antagonism is still controversial. In the present study, when combined with cocaine, CTEP potentiated the hyperlocomotion on spinophilin-KO mice without affecting behavioral sensitization or conditioned place preference, key paradigms to study cocaine addiction in animal models, suggesting that the CTEP effect on KO mice at the dose used may be related mostly to motor aspects, in accordance with previous reports that blockage of mGluR5 produces hyperlocomotion [43, 17]. This potentiated hyperlocomotion could have affected the observation of a lack of behavioral sensitization in the KO mice co-treated with cocaine and CTEP, considering the possibility that the maximal locomotor response could have already been achieved in the initial administrations, not allowing further increases that would characterize a sensitized response.

In the present study, we showed that deletion of spinophilin does not affect acute locomotor effects of cocaine or the conditioned place preference to this drug. However, a loss of spinophilin expression blocks the development of behavioral sensitization to cocaine. This effect was accompanied by blunted cocaine-induced ERK phosphorylation and c-Fos and ∆FosB expression. Therefore, we suggest spinophilin to play an important role in cocaine-induced behavioral sensitization, likely via the activation of ERK1/2 and induction of c-Fos and ∆Fosb in the striatum, a mechanism that may underlie specific processes involved in cocaine addiction. Further studies using an operant-conditioning paradigm for self-administration assessing the effect of spinophilin on reinforcing and motivational effects of cocaine would help elucidating spinophilin’s role in cocaine addiction.

## Abbreviations

AMPA: A-amino-3-hydroxy-5-methyl-4-isoxazolepropionic acid
ANOVA: Analysis of variance
CamKII: Ca2+/calmodulin- dependent protein kinase II
cDNA: Complementary deoxyribonucleic acid
CPP: Conditioned place preference
CTEP: 2-chloro-4-((2,5-dimethyl-1-(4-(trifluoromethoxy)phenyl)-1H-imidazol-4-yl)ethynyl)pyridine
D1R: Dopamine receptor 1
D2R: Dopamine receptor 2
DMSO: Dimethyl sulfoxide
ERK: Extracellular-signal-regulated kinase
KO: Knockout
LTD: Long term depression
LTP: Long term potentiation
mGluR: Metabotropic glutamate receptor
NAc: Nucleus accumbens
NMDA: N-methyl-D-aspartate
PFC: Prefrontal cortex
PKA: Protein kinase A
PP1: Protein phosphatase 1
qPCR: Quantitative polymerase chain reaction
RNA: Ribonucleic acid
SDS-PAGE: Sodium dodecyl sulfate-polyacrylamide gel electrophoresis
VTA: Ventral tegmental area
WT: Wild type

## References

[1] Allen PB, Ouimet CC, Greengard P (1997). Spinophilin, a novel protein phosphatase 1 binding protein localized to dendritic spines. Proc Natl Acad Sci U S A.

[2] Allen PB, Zachariou V, Svenningsson P, Lepore AC, Centonze D, Costa C, Rossi S, Bender G, Chen G, Feng J, Synder GL, Bernardi G, Nestler EJ, Yan Z, Calabresi P, Greengard P (2006). Distinct roles for spinophilin and neurabin in dopamine-mediated plasticity. Neuroscience.

[3] Areal LB, Herlinger AL, Pelicao FS, Martins-silva C, Pires RGW (2017). Crack cocaine inhalation induces schizophrenia-like symptoms and molecular alterations in mice prefrontal cortex. J Psychiatr Res.

[4] Bailey J, Ma D, Szumlinski KK (2012). Rapamycin attenuates the expression of cocaine-induced place preference and behavioral sensitization. Addict Biol.

[5] Borgland SL, Malenka RC, Bonci A (2004). Acute and chronic cocaine-induced potentiation of synaptic strength in the ventral tegmental area: electrophysiological and behavioral correlates in individual rats. J Neurosci.

[6] Bowers MS, McFarland K, Lake RW, Peterson YK, Lapish CC, Gregory ML, Lanier SM, Kalivas PW (2004). Activator of G protein signaling 3: a gatekeeper of cocaine sensitization and drug seeking. Neuron.

[7] Brog JS, Salyapongse A, Deutch AY, Zahm DS (1993). The patterns of afferent innervation of the core and shell in the "accumbens" part of the rat ventral striatum: immunohistochemical detection of retrogradely transported fluoro-gold. J Comp Neurol.

[8] Chiamulera C, Epping-jordan MP, Zocchi A, Marcon C, Cottiny C, Tacconi S, Cosri M, Orzi F, Conquet F (2001). Reinforcing and locomotor stimulant effects of cocaine are absent in mGluR5 null mutant mice. Nat Neurosci.

[9] Evans JC, Robinson CM, Shi M, Webb DJ (2015). The guanine nucleotide exchange factor (GEF) Asef2 promotes dendritic spine formation via Rac activation and spinophilin-dependent targeting. J Biol Chem.

[10] Everitt BJ, Robbins TW (2013). From the ventral to the dorsal striatum: devolving views of their roles in drug addiction. Neurosci Biobehav Rev.

[11] Feng J, Yan Z, Ferreira A, Tomizawa K, Liauw JA, Zhuo M, Allen PB, Ouimet CC, Greengard P (2000). Spinophilin regulates the formation and function of dendritic spines. Proc Natl Acad Sci U S A.

[12] Ferguson SM, Fasano S, Yang P, Brambilla R, Robinson TE (2006). Knockout of ERK1 enhances cocaine-evoked immediate early gene expression and behavioral plasticity. Neuropsychopharmacology.

[13] French SJ, Totterdell S (2003). Individual nucleus accumbens-projection neurons receive both basolateral amygdala and ventral subicular afferents in rats. Neuroscience.

[14] Futter M, Uematsu K, Bullock SA, Kim Y, Hemmings HC, Nishi A, Greengard P, Nairn AC (2005). Phosphorylation of spinophilin by ERK and cyclin dependent PK5 (Cdk5). Proc Natl Acad Sci U S A.

[15] Graybiel AM, Moratalla R, Robertson HA (1990). Amphetamine and cocaine induce drug-specific activation of the c-fos gene in striosome-matrix compartments and limbic subdivisions of the striatum. Proc Natl Acad Sci U S A.

[16] Grossman SD, Futter M, Snyder GL, Allen PB, Nairn AC, Greengard P, Hsieh-Wilson LC (2004). Spinophilin is phosphorylated by Ca2+/calmodulin-dependent protein kinase II resulting in regulation of its binding to F-actin. J Neurochem.

[17] Guimaraes IM, Carvalho TG, Ferguson SSG, Pereira GS, Ribeiro FM (2015). The metabotropic glutamate receptor 5 role on motor behavior involves specific neural substrates. Mol Brain.

[18] Henry DJ, White FJ (1991). Repeated cocaine administration causes persistent enhancement of D1 dopamine receptor sensitivity within the rat nucleus accumbens. J Pharmacol Exp Ther.

[19] Henry DJ, Greene MA, White FJ (1989). Electrophysiological effects of cocaine in the mesoaccumbens dopamine system: repeated administration. J Pharmacol Exp Ther.

[20] Holroyd KB, Adrover MF, Fuino RL, Bock R, Kaplan AR, Gremel CM, Rubinstein M, Alvarez VA (2015). Loss of feedback inhibition via D2 autoreceptors enhances acquisition of cocaine taking and reactivity to drug-paired cues. Neuropsychopharmacology.

[21] Hsieh-Wilson LC, Allen PB, Watanabe T, Nairn AC, Greengard P (1999). Characterization of the neuronal targeting protein spinophilin and its interactions with protein phosphatase-1. Biochemistry.

[22] Hu XD, Huang Q, Yang X, Xia H (2007). Differential regulation of AMPA receptor trafficking by neurabin-targeted synaptic protein phosphatase-1 in synaptic transmission and long-term depression in hippocampus. J Neurosci.

[23] Kalivas PW, Duffy P (1995). D1 receptors modulate glutamate transmission in the ventral tegmental area. J Neurosci.

[24] Kalivas PW, Duffy P (1990). Effect of acute and daily cocaine treatment on extracellular dopamine in the nucleus accumbens. Synapse.

[25] Kelz MB, Nestler EJ (2000). deltaFosB: a molecular switch underlying long-term neural plasticity. Curr Opin Neurol.

[26] Kenny PJ, Boutrel B, Gasparini F, Koob GF, Markou A (2005). Metabotropic glutamate 5 receptor blockade may attenuate cocaine self-administration by decreasing brain reward function in rats. Psychopharmacology.

[27] Kim MJ, Dunah AW, Wang YT, Sheng M (2005). Differential roles of NR2A- and NR2B-containing NMDA receptors in Ras-ERK Signalling and AMPA receptor trafficking. Neuron.

[28] Kumaresan V, Yuan M, Yee J, Famous KR, Anderson SM, Schmidt HD, Pierce RC (2010). Metabotropic glutamate receptor 5 (mGluR5) antagonists attenuate cocaine priming- and cue-induced reinstatement of cocaine seeking. Behav Brain Res.

[29] McClung CA, Nestler EJ (2003). Regulation of gene expression and cocaine reward by CREB and DeltaFosB. Nat Neurosci.

[30] McGeehan AJ, Olive MF (2003). The mGluR5 antagonist MPEP reduces the conditioned rewarding effects of cocaine but not other drugs of abuse. Synapse.

[31] McIntosh S, Howell L, Hemby SE (2013). Dopaminergic dysregulation in prefrontal cortex of rhesus monkeys following cocaine self-administration. Front Psychiatry.

[32] McNamara RK, Logue A, Stanford K, Xu M, Zhang J, Richtand NM (2006). Dose–response analysis of locomotor activity and stereotypy in dopamine D3 receptor mutant mice following acute amphetamine. Synapse.

[33] Morris CW, Watkins DS, Salek AB, Edler MC (2018). The association of spinophilin with disks large-associated protein 3 (SAPAP3) is regulated by metabotropic glutamate receptor (mGluR) 5. Mol Cell Neurosci.

[34] Nader MA, Czoty PW, Gould RW, Riddick NV (2008). Positron emission tomography imaging studies of dopamine receptors in primate models of addiction. Philos Trans R Soc Lond Ser B Biol Sci.

[35] Nestler EJ (2001). Molecular basis of long-term plasticity underlying addiction. Nat Rev Neurosci.

[36] Nestler EJ (2005). The neurobiology of cocaine addiction. Sci Pract Perspect.

[37] Nestler EJ (2008). Transcriptional mechanisms of addiction: role of ΔFosB. Philos Trans R Soc Lond Ser B Biol Sci.

[38] Nygard SK, Klambatsen A, Balouch B, Jenab S (2015). Region and context specific intracellular responses associated with cocaine-induced conditioned place preference expression. Neuroscience.

[39] Parsons LH, Justice JB (1993). Serotonin and dopamine sensitization in the nucleus accumbens, ventral tegmental area, and dorsal raphe nucleus following repeated cocaine administration. J Neurochem.

[40] Pascoli V, Terrier J, Espallergues J, Valjent E, O'Connor EC, Luscher C (2014). Contrasting forms of cocaine-evoked plasticity control components of relapse. Nature.

[41] Pierce RC, Pierce-Bancroft AF, Prasad BM (1999). Neurotrophin-3 contributes to the initiation of behavioral sensitization to cocaine by activating the Ras/mitogen-activated protein kinase signal transduction cascade. J Neurosci.

[42] Ragusa MJ, Dancheck B, Critton DA, Nairn AC, Page R, Peti W (2010). Spinophilin directs protein phosphatase 1 specificity by blocking substrate binding sites. Nat Struct Mol Biol.

[43] Ribeiro FM, Hamilton A, Doria JG, Guimaraes IM, Cregan SP, Ferguson SS (2014). Metabotropic glutamate receptor 5 as a potential therapeutic target in Huntington's disease. Expert Opin Ther Targets.

[44] Ritz MC, Cone EJ, Kuhar MJ (1990). Cocaine inhibition of ligand binding at dopamine, norepinephrine and serotonin transporters: a structure-activity study. Life Sci.

[45] Sakae DY, Marti F, Lecca S, Vorspan F, Morel LJ, Henrion A (2015). The absence of VGLUT3 predisposes to cocaine abuse by increasing dopamine and glutamate signaling in the nucleus accumbens. Mol Psychiatry.

[46] Sarrouilhe D, Tommaso A, Métayé T, Ladeveze V (2006). Spinophilin: from partners to functions. Biochimie.

[47] Satoh A, Nakanishi H, Obaishi H, Wada M, Takahashi K, Satoh K, Hirao K, Nishioka H, Hata Y, Mizoguchi A, Takai Y (1998). Neurabin-II/spinophilin. An actin filament-binding protein with one pdz domain localized at cadherin-based cell-cell adhesion sites. J Biol Chem.

[48] Di Sebastiano AR, Fahim S, Dunn HA, Walther C, Ribeiro FM, Cregan SP, Angers S, Schmid S, Ferguson SSG (2016). Role of Spinophilin in group I metabotropic glutamate receptor endocytosis, signaling, and synaptic plasticity. J Biol Chem.

[49] Sgambato V, Pages C, Rogard M, Besson M, Caboche J (1998). Extracellular signal-regulated kinase (ERK) controls immediate early gene induction on Corticostriatal stimulation. J Neurosci.

[50] Shi X, Miller JS, Harper LJ, Poole RL, Gould TJ, Unterwald EM (2014). Re-activation of cocaine reward memory engages the Akt/GSK3/mTOR signaling pathway and can be disrupted by GSK3 inhibition. Psychopharmacology.

[51] Smith FD, Oxford GS, Milgram SL (1999). Association of the D2 dopamine receptor third cytoplasmic loop with spinophilin, a protein phosphatase-1-interacting protein. J Biol Chem.

[52] Steketee JD, Walsh TJ (2005). Repeated injections of sulpiride into the medial prefrontal cortex induces sensitization to cocaine in rats. Psychopharmacology.

[53] Stephens DJ, Banting G (1999). Direct interaction of the trans-Golgi network membrane protein, TGN38, with the F-actin binding protein, neurabin. J Biol Chem.

[54] Swanson LW (1982). The projections of the ventral tegmental area and adjacent regions: a combined fluorescent retrograde tracer and immunofluorescence study in the rat. Brain Res Bull.

[55] United Nations Office on Drugs and Crime, World Drug Report 2017 (United Nations publication, Sales No. E17XI.6).

[56] Valjent E, Corvol J, Trzaskos JM, Girault A, Hervé D (2006). Role of the ERK pathway in psychostimulant-induced locomotor sensitization. BMC Neurosci.

[57] Vanderschuren L, Di Ciano P, Everitt BJ (2005). Involvement of the dorsal striatum in cue-controlled cocaine seeking. J Neurosci.

[58] Vanhoutte P, Barnier J, Guibert B, Page C, Besson M, Hipskind RA, Caboche J (1999). Glutamate induces phosphorylation of Elk-1 and CREB, along with c -fos activation, via an extracellular signal-regulated kinase-dependent pathway in brain slices. Mol Cell Biol.

[59] Veeneman MM, Broekhoven MH, Damsteegt R, Vanderschuren LJ (2012). Distinct contributions of dopamine in the dorsolateral striatum and nucleus accumbens shell to the reinforcing properties of cocaine. Neuropsychopharmacology.

[60] Volkow ND, Fowler JS, Wang GJ, Baler R, Telang F (2009). Imaging dopamine's role in drug abuse and addiction. Neuropharmacology.

[61] Wang JQ, Fibuch EW, Mao L (2007). Regulation of mitogen-activated protein kinases by glutamate receptors. J Neurochem.

[62] Wang Q, Zhao J, Brady AE, Feng J, Allen PB, Lefkowitz RJ, Greengard P, Limbird LE (2004). Actions in vitro and in vivo at G protein – coupled receptors. Science.

[63] Zhang J, Zhang L, Jiao H, Zhang Q, Zhang D, Lou D, Katz JL, Xu M (2006). C-Fos facilitates the acquisition and extinction of cocaine-induced persistent changes. J Neurosci.

[64] Zhang L, Lou D, Jiao H, Zhang D, Wang X, Xia Y, Zhang J, Xu M (2004). Cocaine-induced intracellular signaling and gene expression are oppositely regulated by the dopamine D1 and D3 receptors. J Neurosci.

[65] Barros VN, Mundim M, Galindo LT, Bittencourt S, Porcionatto M, Mello LE. The pattern of c-Fos expression and its refractory period in the brain of rats and monkeys. Front Cell Neurosci. 2015;9(72):1-8. 10.3389/fncel.2015.00072 PMC4357304 25814929

[66] Martin-Fardon R, Baptista MA, Dayas CV, Weiss F. Dissociation of the effects of MTEP [3-[(2-methyl-1,3-thiazol-4-yl)ethynyl]piperidine] on conditioned reinstatement and reinforcement: comparison between cocaine and a conventional reinforcer. J Pharmacol Exp Ther. 2009;329(3):1084–1090. 10.1124/jpet.109.151357 PMC2683783 19258516

[67] Herzig V, Schmidt WJ. Effects of MPEP on locomotion, sensitization and conditioned reward induced by cocaine or morphine. Neuropharmacology. 2004;47(7):973–984. 10.1016/j.neuropharm.2004.07.037 15555632

[68] Knackstedt LA, Trantham-Davidson HL, Schwendt M. The role of ventral and dorsal striatum mGluR5 in relapse to cocaine-seeking and extinction learning. Addict Biol. 2014; 19(1):87-101. 10.1111/adb.12061 PMC3762937 23710649

[69] Liu ZQ, Gu XH, Yang YJ, Yin XP, Xu LJ, Wang W. D-Serine in the nucleus accumbens region modulates behavioral sensitization and extinction of conditioned place preference. Pharmacol Biochem Behav.2016;143:44-56. 10.1016/j.pbb.2016.02.002 26861675

[70] Fowler MA, Varnell AL, Cooper DC. mGluR5 knockout mice exhibit normal conditioned place-preference to cocaine. Nature Precedings. 2011.

